# Defect in Migration of HSPCs in Nox-2 Deficient Mice Explained by Impaired Activation of Nlrp3 Inflammasome and Impaired Formation of Membrane Lipid Rafts

**DOI:** 10.1007/s12015-024-10775-7

**Published:** 2024-08-13

**Authors:** Kamila Bujko, Mateusz Adamiak, Adrian Konopko, Vira Chumak, Janina Ratajczak, Katarzyna Brzezniakiewicz-Janus, Magdalena Kucia, Mariusz Z. Ratajczak

**Affiliations:** 1 Stem Cell Institute at James Graham Brown Cancer Center, University of Louisville, 500 S. Floyd Street, Rm. 107, Louisville, KY 40202 USA; 2 Center for Preclinical Studies and Technology, Laboratory of Regenerative Medicine at Medical, University of Warsaw, Warsaw, Poland; 3 Laboratory of Regenerative Medicine, Medical University of Warsaw, Warsaw, Poland; 4 Department of Hematology, Multi-Specialist Hospital Gorzow Wlkp, University of Zielona Gora, Zielona Góra, Poland

**Keywords:** Stem cell mobilization, Stem cell homing, Nox2, ROS, Membrane lipid rafts, Nlrp3 inflammasome

## Abstract

NADPH oxidase 2 (Nox2), a superoxide-generating enzyme, is a source of reactive oxygen species (ROS) that regulate the intracellular redox state, self-renewal, and fate of hematopoietic stem/progenitor cells (HSPCs). Nox2 complex expressed on HSPCs associated with several activated cell membrane receptors increases the intracellular level of ROS. In addition, ROS are also released from mitochondria and, all together, are potent activators of intracellular pattern recognition receptor Nlrp3 inflammasome, which regulates the trafficking, proliferation, and metabolism of HSPCs. In the current study, we noticed that Nox2-deficient mice, despite the increased number of HSPCs in the bone marrow (BM), show hematopoietic defects illustrated by delayed recovery of peripheral blood (PB) hematopoietic parameters after sublethal irradiation and mobilize fewer HSPCs after administration of G-CSF and AMD3100. Moreover, Nox2-deficient HSPCs engraft poorly after transplantation into normal syngeneic recipients. To explain these defects at the molecular level, we hypothesized that Nox2-KO decreased ROS level does not efficiently activate Nlrp3 inflammasome, which plays a crucial role in regulating the trafficking of HSPCs. Herein, we report Nox2-deficient HSPCs display i) defective migration to major chemoattractant, ii) impaired intracellular activation of Nlrp3 inflammasome, and iii) a defect in membrane lipid raft (MLRs) formation that is required for a proper chemotactic response to pro-migratory factors. We conclude that Nox2-derived ROS enhances in Nlrp3 inflammasome-dependent manner HSPCs trafficking by facilitating MLRs assemble on the outer cell membranes, and defect in Nox2 expression results in impaired activation of Nlrp3 inflammasome, which affects HSPCs migration.

**Graphical Abstract:**

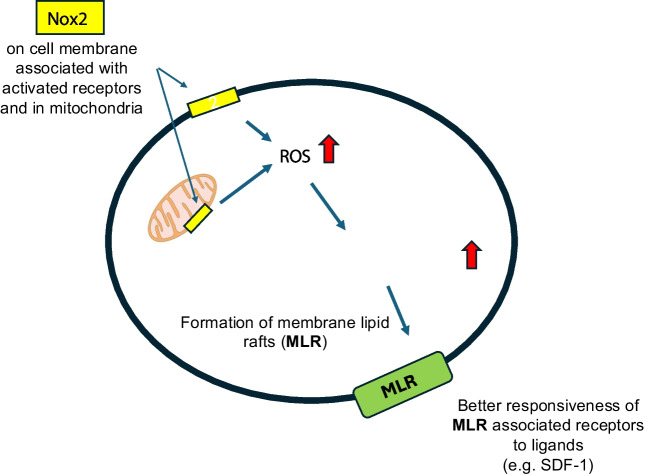

**Supplementary Information:**

The online version contains supplementary material available at 10.1007/s12015-024-10775-7.

## Introduction

NADPH oxidase 2 (Nox2) is a super-oxide generating enzyme that forms reactive oxygen species (ROS) chemically reactive molecules containing oxygen [1–3]. The most critical ROS include peroxides, superoxide, singlet oxygen, and hydroxyl radicals formed as a natural byproduct of the normal metabolism of oxygen. ROS play an essential role in cell signaling and tissue homeostasis, and their level increases in response to environmental stress. Their biological action could depend on the intracellular level and duration being beneficial or detrimental for the cells [1–3]. The most critical ROS is hydrogen peroxide (H_2_O_2_), which also acts as a second messenger, integrating environmental signals and passing them to downstream intracellular signaling cascades [1–3].

In several models, including innate immunity or malignant cells, ROS have been demonstrated to control cell migration [1, 4, 5]. These highly reactive molecules are produced by the cell membrane NADPH oxidase complex and by the mitochondria NAD-dependent oxidoreductases. The assembly of Nox2 subunits on outer cell membranes is facilitated by membrane lipid raft (MLR) clustering to form “Nox2 redox signaling platforms” [6]. It is still unknown if MLRs could also be assembled on mitochondrial membranes.

There are two reasons why we become interested in the role of Nox2 as a source of ROS in the trafficking of hematopoietic stem progenitor cells (HSPCs). First, ROS are involved in the mobilization of HSPCs, as shown, for example, in response to hepatocyte growth factor (HGF) by activating mTOR-FOXO3a [7] and in response to sphingosine-1 phosphate (S1P) by promoting the release of stromal-derived factor-1 (SDF-1) [8]. Another important reason was that ROS activates the Nlrp3 inflammasome, which plays, as we demonstrated recently, a crucial role in the trafficking of HSPCs [6]. To support this, we reported that Nlrp3 inflammasome-deficient mice are poor mobilizers and engraft poorly with normal bone marrow-derived mononuclear cells (BMMNCs). Thus, we have hypothesized that Nox2-ROS deficiency will impact the trafficking of HSPCs due to a defect in activating the Nlrp3 inflammasome. Moreover, this defect at the molecular level could result in impaired formation of MLRs in Nox2-deficient animals. MLRs, as reported, play a crucial role in signaling from “raftophilic receptors” that regulate the trafficking and biological responses of HSPCs to chemoattractants, growth factors, and cytokines.

We report herein that the defect in the trafficking of Nox2-deficient mice HSPCs is explained by impaired activation of Nlrp3 inflammasome and MLRs assembly.

## Material and Methods

### Animals

Pathogen-free, 6–8-week-old female B6.129S-Cybb^tm1Din^/J (Nox2-KO) mice were purchased from the Jackson Laboratory (Bar Harbor, ME; USA) at least two weeks before experiments. C57BL/6 J (WT) mice were purchased from the Central Laboratory for Experimental Animals, the Medical University of Warsaw, and the Jackson Laboratory (Bar Harbor, ME; USA). Animal studies were approved by the Animal Care and Use Committee of the University of Louisville (Louisville, KY, USA) and the Warsaw Medical University (Warsaw, Poland).

### Transwell Migration Assay

In the Transwell migration assay, aliquots of WT and Nox2-KO BM-derived MNCs (1 × 10^6^/100 µl) were added to the upper chamber (5 µm pore filters) of a Costar Transwell 24-well plate (Corning Costar, New York, NY, USA). The lower chamber contained RPMI-1640 assay medium (650 μl) plus 0.5% BSA (control) or the medium supplemented with SDF-1 (100 ng/ml), ATP (10 μM) or S1P (0.1 μM). In some experiments, nigericin (10 μM) was added to chemoattractants, and in others, the lower chamber contained SDF-1 (5 ng/ml), or LL-37 (2.5 μg/ml), or both SDF-1 and LL-37. The plate was incubated for 3 h at 37 °C and 5% CO_2_. Following incubation, aliquots of migrated cells from the lower chambers were resuspended in a methylcellulose base medium (R&D Systems, Minneapolis, MN, USA), supplemented with murine recombinant granulocyte/macrophage colony-stimulating factor (GM-CSF) (25 ng/ml) and recombinant murine interleukin 3 (IL-3) (10 ng/ml) (PeproTech, Rocky Hill, NJ, USA). After 7 days of culture (37 °C and 5% CO_2_), CFU-GM colonies were counted under an inverted microscope.

### In VivoMobilization Studies

For mobilization studies, WT and Nox2-KO mice were injected subcutaneously (SC) with Granulocyte-Colony Stimulating Factor (G-CSF, 150 μg/kg daily; Amgen, Thousand Oaks, CA, USA) for 3 days or with a single dose of AMD3100 (Sigma-Aldrich, St. Louis, MO, USA) [5 mg/kg, intraperitoneally (IP)]. 6 h after the last G-CSF injection and 1 h after AMD3100 injection, mice were bled from the retro-orbital plexus for white blood cells count (WBCs) analysis, and peripheral blood (PB) was obtained from the vena cava (with a 25-gauge needle and 1-ml syringe containing EDTA). Total nucleated cells (TNCs) were isolated by hypotonic lysis of red blood cells (RBCs) in 1 × BD Pharm Lyse buffer (BD Biosciences, San Jose, CA, USA) and further analyzed for SKL cells and CFU-GM clonogenic progenitors as described below.

### Peripheral Blood WBC Count

To obtain white cell counts, 50 μl of PB was taken from the retro-orbital plexus of mice into microvette EDTA-coated tubes (Sarstedt Inc., Newton, NC, USA) and run on a HemaVet 950FS hematology analyzer (Drew Scientific Inc., Oxford, CT, USA) or Exigo hematology analyzer (Boule Diagnostics, Spanga, Sweden) within 2 h of collection.

### FACS Analysis

For Fluorescence-activated cell sorting analysis of SKL cells (Sca-1^+^c-Kit^+^Lin^−^), PB-derived TNCs were stained with the following monoclonal antibodies: rat anti-CD45R/B220 (phycoerythrin [PE]; clone RA3-6B2), anti-Gr-1 (PE; clone RB6-8C5), anti-TCRαβ (PE; clone H57–597), anti-TCRγδ (PE; clone GL3), anti-CD11b (PE; clone M1/70), anti-Ter119 (PE; clone TER-119), and anti-Ly-6A/E (Sca-1; APC; clone E13–161.7) and anti-CD117 (c-Kit, FITC; clone 2b8). Staining was performed in RPMI-1640 medium containing 2% fetal bovine serum (FBS). All monoclonal antibodies were added at saturating concentrations, and the cells were incubated for 30 min on ice, washed twice, and analyzed on LSR II or FACS Verse cytometer (BD Biosciences, San Jose, CA, USA) as described [8].

### Sorting Strategy for SKLs from Murine Bone Marrow

Sca-1^+^c-Kit^+^Lin^−^ cells (SKLs) were isolated from the BM of 6-week-old WT and Nox2-KO mice. Bone marrow (BM) was flushed from the cavities of the tibias and femurs. The population of TNCs was obtained after the lysis of RBCs using 1 × BD Pharm Lyse buffer (BD Biosciences, San Jose, CA, USA). TNCs were resuspended in RPMI-1640 medium containing 2% FBS. Staining for SKLs was performed as described above. The cells were subsequently washed, resuspended in medium plus 2% FBS, and sorted using a MoFlo Astrios EQ cell sorter (Beckman Coulter, Brea, CA, USA) as reported in the past [9].

### Clonogenic CFU-GM and BFU-E Assays

BMMNCs and/or peripheral blood mononuclear cells (PBMNCs) were resuspended in a methylcellulose base medium (R&D Systems, Minneapolis, MN, USA). The medium for clonogenic CFU-GM assays was supplemented with 25 ng/ml GM-CSF and 10 ng/ml mIL-3. To perform burst-forming unit-erythroid (BFU-E) assays, cell samples were suspended in methylcellulose supplemented with erythropoietin (EPO, 5 U/ml) and IL-3 (10 ng/ml; PeproTech, Rocky Hill, NJ, USA). Cells were incubated for 7 days (37 °C, 5% CO_2_) for CFU-GM and 14 days for BFU-E assays. As reported, the CFU-GM and BFU-E colonies were scored using a simple inverted microscope (Olympus, Center Valley, PA, USA) [8, 9].

### Evaluation of Hematopoietic Stem/Progenitor Cell Mobilization

In the assessment of cell mobilization, WBCs, SKL cells, and the number of CFU-GM colonies were determined according to the above protocols. For recalculation of SKLs and circulating colony-forming unit-granulocyte/macrophage CFU-GM number in PB following formulas were used: (number of white blood cells [WBCs] × number of CFU-GM colonies)/number of WBCs plated = number of CFU-GM per ml of PB, and (number of WBCs × number of SKL cells)/number of gated WBCs = number of SKL cells per μl of PB as reported in the past [10].

### Short-Term Homing Experiments

Experimental mice (WT, Nox2-KO, or WT treated with N-acetyl-l-cysteine (NAC) (150 mg/kg, IP, for 5 days) were irradiated with a lethal dose of γ-irradiation (10 Gy). After 24 h, the animals were transplanted (by tail vein injection) with 5 × 10^6^ BM cells from WT mice alone, Nox2-KO, or WT treated with NAC (0.5 µM for 1 h). All cells were labeled with PKH67 Green Fluorescent Cell Linker (Sigma-Aldrich, St Louis, MO, USA) according to the manufacturer’s protocol. 24 h after transplantation, BM cells from the femurs were isolated via Ficoll–Paque centrifugation and divided into two aliquots. One aliquot of cells was analyzed on a flow cytometer for PKH67-positive cells, and the second aliquot of cells was plated in methylcellulose cultures and stimulated to grow CFU-GM colonies with GM-CSF (25 ng/ml) and IL-3(10 ng/ml). After 7 days of incubation (37 °C and 5% CO_2_), the number of colonies was scored under an inverted microscope as described [6, 11].

### Evaluation of Engraftment

For engraftment experiments, mice (WT, Nox2-KO, or WT treated with NAC [150 mg/kg, IP, for 5 days]) were irradiated with a lethal dose of γ-irradiation (10 Gy). After 24 h, the animals were transplanted (by tail vein injection) with 1.5 × 10^5^ BM cells from WT mice alone, Nox2-KO, or WT treated with NAC (0.5 µM for 1 h). The femora of transplanted mice were flushed with phosphate-buffered saline (PBS) on day 12 post-transplant. Purified via Ficoll-Paque, BM cells were plated in methylcellulose cultures and stimulated to grow CFU-GM colonies with G-CSF (25 ng/ml) plus IL-3 (10 ng/ml). The colonies’ number was scored under an inverted microscope after 7 days of incubation (37 °C and 5% CO2). Spleens were also removed and fixed in Fekete’s solution for CFU-S assays, and colonies were counted on the surface of the spleen, as reported [6, 11].

### Recovery of Leukocytes and Platelets

Experimental mice (WT, Nox2-KO, or WT treated with NAC [150 mg/kg, IP, for 5 days]) were irradiated with a lethal dose of γ-irradiation (10 Gy). After 24 h, the animals were transplanted (by tail vein injection) with 1.0 × 10^6^ BM cells from WT mice alone, Nox2-KO, or WT treated with NAC (0.5 µM for 1 h). Transplanted mice were bled from the retro-orbital plexus at various intervals to obtain WBC and platelet counts (PLTs) samples. Briefly, 50 μl of PB were drawn into EDTA-coated Microvette tubes (Sarstedt Inc., Newton, NC, USA) and run within 2 h of collection on Exigo hematology analyzer (Boule Diagnostics, Spanga, Sweden).

### Membrane Lipid Rafts (MLRs) Detection

Murine tibias and femurs from WT and Nox2-KO animals were flushed, and murine SKL cells were purified using FACS, as described above. Next, WT and Nox2-KO isolated SKL cells were plated on fibronectin-coated plates overnight, then incubated with SDF-1 (50 ng/ml) and LL-37 (2.5 μg/ml), then washed and fixed in 3.7% paraformaldehyde. Nox2-KO BM SKL cells were exposed to 1 µM PGE2, washed, and fixed in 3.7% paraformaldehyde. The cholera toxin B subunit conjugated with FITC (Sigma-Aldrich, St. Louis, MO, USA) was applied to detect the ganglioside GM1, and rat monoclonal anti-CXCR4 IgG antibody (R&D Systems, Minneapolis, MN, USA) and Alexa Fluor 594 goat anti-rat IgG antibody (Invitrogen, Waltham, MA, USA) was applied to detect CXCR4. The stained cells were examined, and images were generated using a FluoView FV1000 laser-scanning confocal microscope (Olympus America Inc., Center Valley, PA, USA) as described previously [6, 12].

### The Activity of Reactive Oxygen Species (ROS)

BMMNCs were isolated from femurs and tibias of WT and Nox2-KO mice. Briefly, 2 × 10^6^ cells were incubated with the cell-permeant reagent 2’,7’–dichlorofluorescein diacetate (DCFDA) according to manufacture protocol (DCFDA/H2DCFDA-Cellular ROS Assay Kit, Abcam, Cambridge, MA, USA), washed and stimulated with 1 µM PGE2 for 4 h. After the incubation, cells were washed and resuspended in RPMI-1640 medium containing 2% FBS and analyzed by FACS for the formation of fluorescent dichlorofluorescein (DCF). At least 1 × 10^5^ cells were counted to determine the activity of reactive oxygen species (ROS).

### Oxygen Consumption Rate (OCR) Measurements on Lin- BMMNC

Bone marrow stem cells were isolated from WT and Nox-2-KO mice. Lin^−^ cells were purified from the bone marrow of WT and Nox-2-KO mice using a Direct Lineage Cell Depletion Kit (Miltenyi Biotec, Bergisch Gladbach, Germany). Briefly, cells were incubated for 10 min at 4 °C with MicroBeads conjugated to monoclonal antibodies against CD5, CD11b, CD45R (B220), Anti-Gr-1 (Ly-6G/C), 7–4, and Ter-119 (Direct Lineage Cell Depletion Kit, Miltenyi Biotec, Bergisch Gladbach, Germany). Following incubation, the cells were washed with 3 ml of phosphate-buffered saline (PBS) and centrifuged at 1800 rpm for 10 min at 4 °C. The cell pellet was resuspended in 1 ml of PBS, and the cell suspension was applied to an LC column (Miltenyi Biotec, Bergisch Gladbach, Germany) placed in a magnetic field (Manual Cell Separator, Miltenyi Biotec, Bergisch Gladbach, Germany) after rinsing the column with 3 ml of PBS. The column was then washed with 3 ml of PBS to elute and collect the purified Lin^−^ cells.

Following the manufacturer’s protocol, the oxygen consumption rate was measured at 37 °C using a Seahorse XF HS MINI (Agilent Technologies, Santa Clara, CA, United States) with the Cell Mito Stress Test Kit. Briefly, 400,000 cells per well in 180 μl of phenol-red-free RPMI media were added to a Seahorse XFp PDL miniplate and incubated at 37 °C without CO_2_ for 1 h. OCR measurements were taken from a 90% confluent monolayer culture. A protocol was followed to assess mitochondrial function indices, involving sequential injections of oligomycin, FCCP, and a mixture of rotenone and antimycin A through the ports of Seahorse Flux Pak cartridges. The final concentrations of these compounds were 1.5 μM, 2 μM, and 0.5 μM, respectively, enabling the determination of basal and maximal respiratory.

### Real-Time RT-PCR

MNCs were isolated from WT and Nox2-KO mice’s BM and lysed using 1 × BD Pharm Lyse buffer (BD Biosciences, San Jose, CA, USA). Then, total RNA was isolated using the RNeasy Mini kit, including treatment with DNase I (Qiagen, Hilden, Germany). According to the manufacturer’s recommendations, cDNA was prepared from an RNA template using the First Strand cDNA Synthesis Kit (Thermo Fisher Scientific, Waltham, MA, USA). Real-time PCR was performed using ABI Prism 7500 sequence detection system (Applied Biosystems, Carlsbad, CA, USA) with the use of SYBR Green PCR Master Mix reagents (Applied Biosystems, Carlsbad, CA, USA) and specific primers listed in Supplementary Table 1. All PCRs were performed using the following conditions: pre-denaturation at 95 °C for 3 min, 40 cycles of denaturation at 95 °C for 15 s and annealing at 60 °C for 30 s. A melting curve was generated to confirm primer specificity and to avoid the possibility of amplifying DNA contamination. The relative quantity of a target gene, normalized to the β2-microglobulin gene as the endogenous control and relative to a calibrator, was expressed as 2–ΔΔCt (fold difference).

### Statistical Analysis

Statistical analysis was performed using GraphPad Prism 9.0 (GraphPad Software Inc., La Jolla, CA, USA). All data are provided as an average ± SD. Statistical data analysis was performed using multiple unpaired t-tests. In all calculations, p ≤ 0.05 was considered significant.

## Results

### Nox2-KO Mice have more HSPCs in BM but Show Defective PB Cell Count Recovery After Sublethal Irradiation

**Fig. 1:**
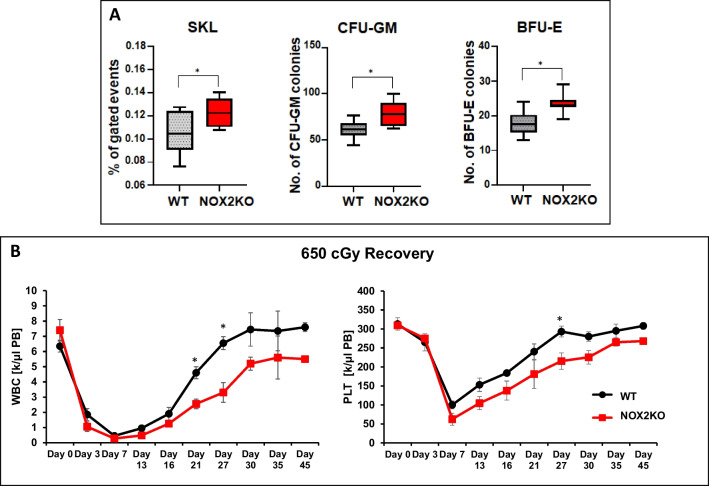
Delayed recovery of peripheral blood counts in Nox2 mice despite increased number of HSPCs in mutant animals. **A** The number of SKL cells, CFU-GM, and BFU-E clonogenic progenitors obtained in steady state conditions from BMMNCs samples from WT and Nox2-KO mice. FACS detected SKL cells after staining with appropriate antibodies. BMMNCs were cultured on human methylcellulose medium supplemented with GM-CSF and IL-3 to grow CFU-GM colonies and EPO and IL-3 to grow BFU-E colonies. Colonies were counted using a simple inverted microscope after 7 (CFU-GM) and 14 (BFU-E) days of culturing. *n* = 6 mice in each group, data represent means ± SD; *p ≤ 0.05. **B** Recovery of WBC and PLT counts in sublethal irradiated (650 cGy) WT and Nox2-KO mice. *n* = 6 mice in each group, data represent means ± SD; *p ≤ 0.05

Nox2 deficiency leads to an increase in the number of HSPCs in BM [4]. Our data confirmed that Nox2-KO mice under steady-state conditions have a significantly higher number of SKL cells and clonogenic potential than control WT mice (Fig. 1A). However, to our surprise, we noticed delayed recovery of PB cell counts in these animals after sublethal (650 cGy) irradiation (Fig. 1B). This indicates a “hidden” hematopoietic defect in Nox2-KO mice that becomes visible under stress.

### Nox2-KO HSPCs Show Impaired Chemotaxis to BM-chemoattractant Due to Defective Activation of Nlrp3 Inflammasome and Impaired MLRs Assembly

**Fig. 2:**
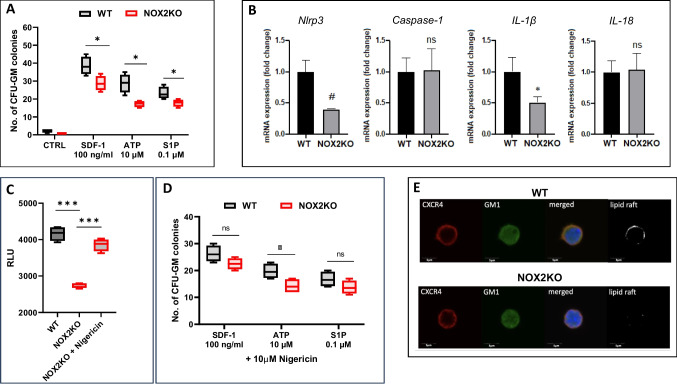
Defect in migration of HSPCs from Nox2-KO mice explained by a defect in MLRs formation. **A** Nox2-KO BMMNCs show decreased chemotactic responsiveness to major BM chemoattractants. Chemotactic responsiveness was determined by counting the number of CFU-GM colonies formed from the WT and Nox2-KO BMMNCs migrated to the medium supplemented with SDF-1 (100 ng/ml) or S1P (0.1 μM) or ATP (10 μM) in Transwell migration assay. Results are combined from two independent experiments and showed as mean ± SD; *p ≤ 0.05. **B** Expression of Nlrp3, caspase-1, IL-1β, and IL-18 mRNAs in BMMNCs samples obtained from WT and Nox2-KO mice measured by qRT-PCR. Results of qRT-PCR were normalized to the β2 microglobulin (β2m) expression levels; the comparative ΔCT method was employed to evaluate relative expression. Results are combined from three independent BMMNC isolations and analyses and presented as mean ± SD.*p ≤ 0.05, #*p* ≤ 0.005. **C** Nox2-KO SKL cells show a decrease in the Nlrp3 inflammasome activation. SKL cells were isolated from WT and Nox2-KO animals, and Nlrp3 inflammasome activation was assessed by Caspase-Glo® 1 Inflammasome assay (Promega). Additionally, Nox2-KO SKL cells were stimulated by 10 µM nigericin for 4 h. Results are combined from two separate measurements and presented as mean ± SD; ***p ≤ 0.001 **D** Nlrp3 inflammasome activation by nigericin increases chemotactic responsiveness of Nox2-KO BMMNCs. Chemotactic responsiveness was determined by counting the number of CFU-GM colonies formed from the WT, and Nox2-KO BMMNCs migrated to the medium supplemented with nigericin (10 μM) and SDF-1 (100 ng/ml) or S1P (0.1 μM) or ATP (10 μM) in Transwell migration assay. Results are combined from two independent experiments and showed as mean ± SD; *p ≤ 0.05. **E** Defect in lipid raft formation in Nox2-KO HSPCs. BM-purified SKL cells were exposed to LL-37 (2.5 µg/ml) and SDF-1 (50 ng/ml), stained with lipid raft marker—cholera toxin subunit B (GM1, FITC), rat anti-mouse CXCR4, and secondary anti-rat antibody (Alexa Fluor 594). Confocal analysis showed lipid raft formation in WT-derived SKL cells (top panel) but not in Nox2-KO BM-derived SKL cells (lower panel). Representative images, from two separate isolations and staining are shown

Based on published data that Nox2 generated reactive oxygen species (ROS) may promote migration of HSPCs to stromal-derived factor-1 (SDF-1) gradient [4, 6], we performed chemotaxis assays with BMMNC to SDF-1 and in addition for the first time to two other supportive BM chemotactic factors for HSPCs: sphingosine-1 phosphate (S1P) and extracellular ATP (eATP). [11, 13] As demonstrated in Fig. 2A, we noticed defective migration of Nox2-deficient CFU-GM not only to SDF-1 but also to S1P and eATP.

Since, as we have reported, Nlrp3 inflammasome plays an important role in HSPCs migration [6], we evaluated the mRNA level expression of significant components of Nlrp3 inflammasome in BMMNC of Nox2-KO mice. We compared this data to BMMNC isolated from control WT animals (Fig. 2B). We noticed a ~ 50% reduction of mRNA expression for Nlrp3 and IL-1β. At the same time, we did not observe differences in mRNA expression for IL-18 and caspase-1. Additionally, we observed Nox2-KO BMMNC a reduction in mRNA expression for SDF-1 and CXCR4 compared to control animals (Supplementary Fig. 1A). Defective activation of Nlrp3 inflammasome was subsequently demonstrated by employing Glow assay, which could be explained by the lower generation of ROS in Nox2-deficient cells (Supplementary Fig. 1B). This corresponded with **lower** mitochondrial activity of Lin^−^ cells from Nox-2-KO mouse BM compared to WT control animals. Supplementary Fig. 2B compares oxygen consumption rate (OCR) in cells isolated from WT and Nox-2-KO mice, and Supplementary Fig. 2A compares basal and maximum respiration between BM cells isolated from WT and Nox-2-KO mice.

The activation of Nlrp3 inflammasome in Nox2-KO HSPCs was enhanced after exposure to direct activation of Nlrp3 inflammasome by nigericin (Fig. 2C). More importantly, the chemotactic responsiveness of HSPCs to SDF-1 and S1P gradients was also improved, except in the presence of this compound. However, in the case of eATP, this improvement was not statistically significant (Fig. 2D).

Next, since the responsiveness of HSPCs to SDF-1 gradient may be sensitized/enhanced by enhancing the incorporation of CXCR4 into MLRs as demonstrated in the presence of innate immunity mediator LL-37 (cathelicidin) [14], we tested the responsiveness of Nox2-deficient and normal WT clonogenic progenitors CFU-GM to a low dose of SDF-1 in the presence or absence of LL-37. We learned from this experiment that Nox2-deficient cells do not increase their responsiveness to the SDF-1 + LL-37 gradient, which suggests a defect in MLR formation (Supplementary Fig. 3). Based on this, we evaluated MLR formation directly by employing confocal analysis in WT and Nox2-KO sorted Sca-1^+^c-Kit^+^Lin^−^ (SKL) cells and noticed that mutant cells, in contrast to WT mice-derived cells have a defect in assembling MLRs in the cell membrane in response to SDF-1 + LL-37 (Fig. 2E).

It has been reported that prostaglandin E2 (PGE2) stimulates Nlrp3 inflammasome in ROS dependent manner [15–17]. Therefore, we evaluated the effect of PGE2 stimulation of murine BMMNC on mRNA expression for CXCR4, SDF-1, and Nlrp3 inflammasome components. Normal BMMNC were stimulated with PGE2 alone or in the presence of ROS scavenger, N-acetyl-l-cysteine (NAC). We noticed that PGE2 enhanced the expression of all these genes in a ROS-dependent manner, and ROS inhibition by NAC attenuated this effect (Supplementary Fig. 4A). In addition, Supplementary Fig. 4B demonstrates the negative effect of NAC as a ROS scavenger on PGE2-induced MLR formation on murine SKL cells confirmed by direct confocal analysis.

### Nox2-KO Mice are Poor Mobilizers, and Direct Activation of Nlrp3 Inflammasome by Nigericin Ameliorates this Defect

**Fig. 3:**
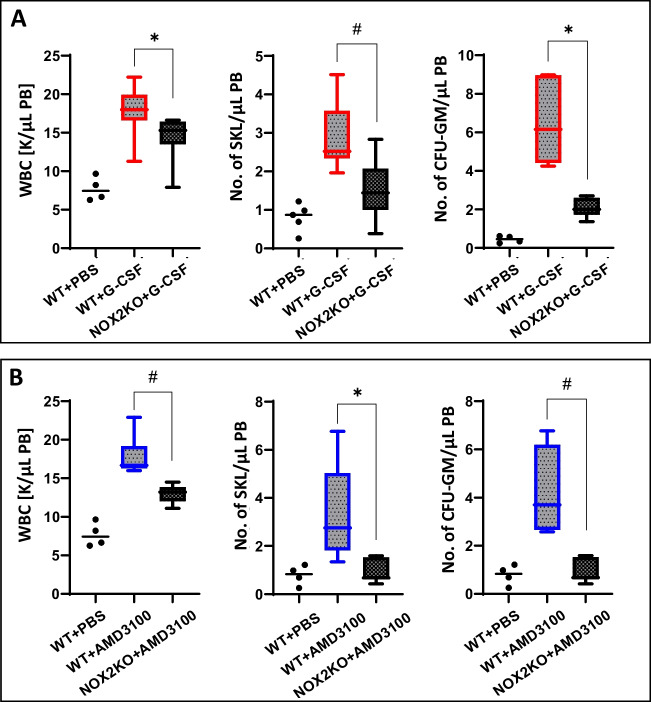
Nox2-KO mice are poor G-CSF and AMD3100 mobilizers. For mobilization studies, WT and Nox2-KO mice were mobilized for 3 days with G-CSF at 150 μg/kg per day by subcutaneous injection (SC) (**A**) and by a single dose of AMD3100 at 5 mg/kg in intraperitoneal injection (IP) (**B**). 6 h after the last G-CSF injection and 1 h after AMD3100 injection, mice were bled from the retro-orbital plexus for WBCs analysis, and PB was obtained from the vena cava for FACS analysis of SKL cells and CFU-GM clonogenic progenitors assay. *n* = 4 for control (WT + PBS) group and *n* = 6 for experimental groups. The data are presented as means ± SD. Unpaired Student’s t-test was used to determine significance (*p ≤ 0.05, #*p* ≤ 0.005)

**Fig. 4:**
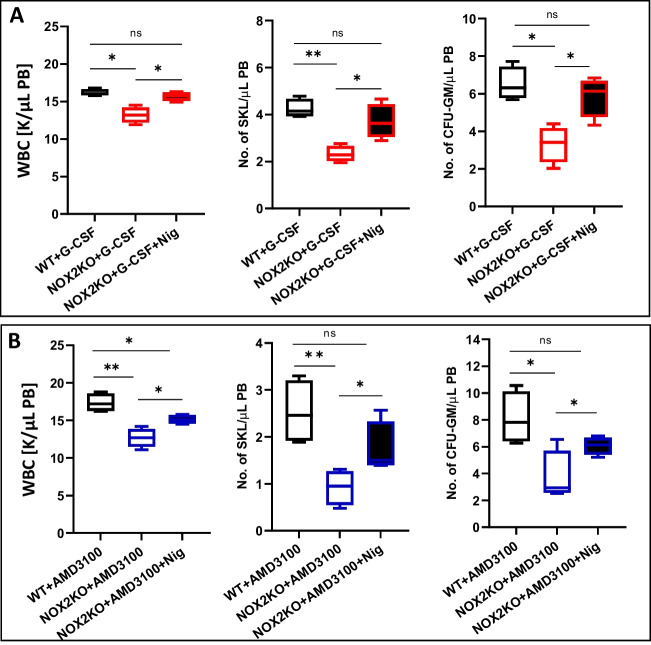
Supplementation with Nlrp3 inflammasome activating nigericin ameliorates mobilization defect in Nox2-KO mice. WT and Nox2-KO mice were mobilized for 3 days with G-CSF at 150 μg/kg per day by subcutaneous injection (SC) (**A**) and by a single dose of AMD3100 at 5 mg/kg in intraperitoneal injection (IP) (**B**). Additionally, mice received the IP injection of nigericin at 0.5 mg/kg before each injection of mobilizing agent. 6 h after the last G-CSF injection and 1 h after AMD3100 injection, mice were bled from the retro-orbital plexus for WBCs analysis. PB was obtained from the vena cava for FACS analysis of SKL cells and CFU-GM clonogenic progenitors assay. *n* = 6 animals per group, the data are presented as means ± SD. Multiple unpaired t-tests were used to determine significance (*p ≤ 0.05; **p ≤ 0.01)

Nlrp3 inflammasome, as reported previously, is involved in promoting the mobilization of HSPCs [18]. Moreover, patients with chronic granulomatous disease (CGD), an inherited disorder caused by either Nox2 deficiency or mutations in the NADPH oxidase complex subunits, are poor mobilizers of HSPCs [19]. To address this issue better, we performed mobilization studies in Nox2-KO mice. These mice, as well as normal control animals, were mobilized with G-CSF or AMD3100. We noticed that Nox2 deficient mice had a lower number of WBC, SKL cells, and, in particular, clonogenic CFU-GM progenitors in PB after G-CSF (Fig. 3A) and AMD3100 (Fig. 3B) exposure. Thus, this defect in mice somehow recapitulates the defect observed in CGD patients [4, 19]. Since Nox2-KO mice show defective ROS-mediated activation of Nlrp3 inflammasome, we exposed mice before mobilization to Nlrp3 inflammasome stimulating agent nigercin. We noticed significant improvement of both G-CSF (Fig. 4A) and AMD3100 (Fig. 4B) induced egress of HSPCs from BM into PB, confirming an important role for Nlrp3 inflammasome activation in the mobilization process.

**Fig. 5:**
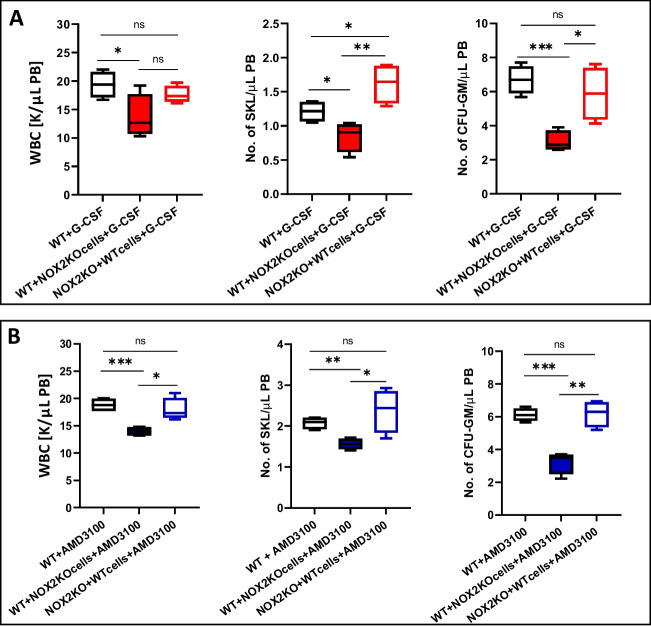
Mobilization studies in Nox2 – WT chimeric mice. Nox2-KO BM cells were transplanted into WT mice, and WT BM cells into Nox2-KO recipients. Transplantation of WT cells into WT recipients served as a control for these experiments. After 6 weeks of cell transplant, hematological chimeras were mobilized for 3 days with G-CSF at 150 μg/kg per day by subcutaneous injection (SC) (**A**) and by a single dose of AMD3100 at 5 mg/kg in intraperitoneal injection (IP) (**B**). 6 h after the last G-CSF injection and 1 h after AMD3100 injection, mice were bled from the retro-orbital plexus for WBCs analysis, and PB was obtained from the vena cava for FACS analysis of SKL cells and CFU-GM clonogenic progenitors assay. *n* = 6 animals per group, the data are presented as means ± SD. Multiple unpaired t-tests were used for the determination of significance (*p ≤ 0.05; **p ≤ 0.01; *** *p* ≤ 0.001)

Next, to address if defective mobilization in Nox2 deficient mice depends on lack of Nox2 in HSPCs or BM hematopoietic microenvironment, we created irradiation chimeras by generating WT mice carrying Nox2 hematopoiesis and Nox2-KO mice reconstituted with WT BMMNCs (Fig. 5). Both WT animals and chimeric mice were mobilized by G-CSF (Fig. 5A) or AMD3100 (Fig. 5B). We observed mobilization defects in chimeric WT mice reconstituted with Nox2-KO BMMNCs. This implicates stem cell-mediated defect in Nox2-deficiency.

### WT BMMNC Exposed to ROS Inhibitors Shows Defective Homing and Engraftment in WT Mice, and WT Mice Exposed to ROS Inhibitors Engraft Worse with WT BMMNC

Since Nox2-KO HSPCs show defective migration to major BM homing chemoattractants (Fig. 1A), we focused first on ROS inhibition by N-acetyl-l-cysteine (NAC) in BMMNC on the ability of these cells to home and engraft after transplantation into WT recipients. Compared with WT control cells, we found that the number of fluorochrome PKH67-labeled donor cells was reduced by ~ 25% in the BM of transplanted animals 24 h after injection with NAC-exposed BMMNCs. Similarly, the number of donor-derived CFU-GM colonies in BM after injection of NAC-exposed BMMNCs was reduced by more than 20% relative to control WT BMMNCs (Supplementary Fig. 5A – upper panel). Moreover, in short-term engraftment experiments, we observed that, 12 days after transplantation of NAC-exposed cells, the numbers of donor-derived CFU-S progenitors in the spleen and CFU-GM progenitors in BM were reduced by ~ 25% (Supplementary Fig. 5A – middle panel). Corresponding to these results, we observed that mice transplanted with NAC-exposed cells had slower kinetics of recovery of leukocytes and platelets (Supplementary Fig. 5A – lower panel).

We subsequently observed similar results when NAC inhibited BM-induced ROS after irradiation conditioning for transplantation in recipient mice. Accordingly, the number of fluorochrome-labeled donor-derived cells was reduced by ~ 20% in the BM of NAC-exposed mice (Supplementary Fig. 5B upper panel). Similarly, the number of donor WT CFU-GM clonogenic progenitors in NAC-exposed BM was reduced by more than 20% relative to control WT BMMNCs. Moreover, in short-term engraftment experiments, we observed that, 12 days after transplantation of WT cells into NAC-exposed recipients, the numbers of donor-derived CFU-GM progenitors in BM and CFU-S progenitors in the spleen were reduced by more than 20% (Supplementary Fig. 5B middle panel). Corresponding with these results, we observed that NAC-exposed mice transplanted with WT cells had slower recovery kinetics of leukocytes and platelets (Supplementary Fig. 5B lower panel).

### Homing and Engraftment Studies Employing Nox2 Mutant Mice

**Fig. 6:**
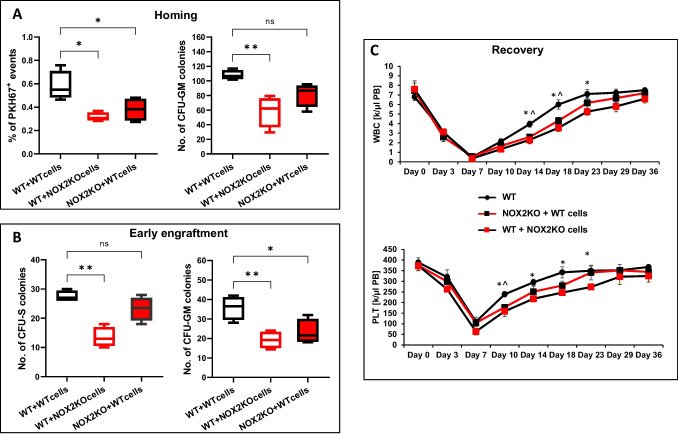
Transplantation studies with WT and Nox2-KO BMMNCs into WT and Nox2-KO animals. **A** Nox2-KO BMMNCs show defective homing into BM after transplantation. Lethally irradiated WT and Nox2-KO mice were transplanted with PKH67 immunofluorescence cell linker labeled BMMNCs from Nox2-KO and WT mice, respectively. 24 h after transplantation, the number of PKH67 + cells in the femoral bone was evaluated by FACS, and the CFU-GM clonogenic progenitors were enumerated in an in vitro colony assay. *n* = 6 mice in each group, the data are presented as means ± SD; *p ≤ 0.05. **B** Nox2-KO BMMNCs show defective early engraftment into BM after transplantation. Lethally irradiated WT and Nox2-KO mice were transplanted with BMMNCs from Nox2-KO and WT mice. 12 days after transplantation, femoral BMMNCs were harvested and plated to count the CFU-GM colonies. The spleens were removed and fixed in Fekete’s solution to count the CFU-S colonies. *n* = 6 mice in each group, the data are presented as means ± SD; *p ≤ 0.05; **p ≤ 0.01. **C** Nox2-KO BMMNCs show defects in hematological reconstitution after transplantation**.** Lethally irradiated WT and Nox2-KO mice were transplanted with BMMNCs from Nox2-KO and WT mice. White blood cells (WBC) and platelets (PLT) were counted at intervals (at 0, 3, 7, 10, 14, 18, 23, 29 and 36 days after transplantation). *n* = 6 mice in each group, the data are presented as means ± SD; *p ≤ 0.05 for the group WT + WT cells vs. WT + Nox2-KO cells; ^ *p* ≤ 0.05 for the group WT + WT cells vs. Nox2-KO + WT cells. WT animals transplanted with WT-derived BMMNCs served as a control in all experiments

Finally, to confirm data with NAC in homing and engraftment of HSPCs address, we transplanted Nox2-KO BMMNCs into WT mice and WT BMMNCs into Nox2-KO recipients. Transplantation of WT cells into WT recipients served as a control for these experiments (Fig. 6). Figure 6A shows the number of donor PKH67 labeled cells present 24 h after transplantation and the number of clonogenic donor-derived CFU-GM present in the BM of the recipient mice. Figure 6B shows the number of donor-derived CFU-S in spleens and CFU-GM in BM 12 days after transplantation in recipient mice. Figure 6C demonstrates recovery of WBC and PLT counts after transplantation of donor cells into recipient mice. This data revealed a profound defect of homing, early engraftment, and recovery of PB counts in Nox2 cells transplanted into WT animals compared to control transplantations of WT cells into WT animals. This again indicates that Nox2 deficiency in HSPCs was a major driver of this defect.

**Fig. 7:**
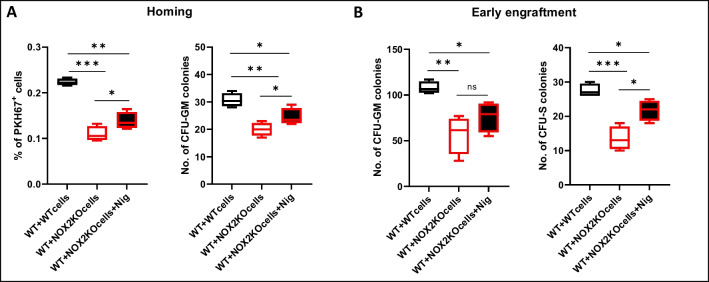
Supplementation with Nlrp3 inflammasome activating nigericin ameliorates engraftment defect of Nox2-KO BMMNCs in WT mice. **A** Lethally irradiated WT mice were transplanted with PKH67 immunofluorescence cell linker labeled BMMNCs from WT animals, Nox2-KO mice, and Nox2-KO mice with additional cell treatment with nigericin. 24 h after transplantation, the number of PKH67 + cells in the femoral bone was evaluated by FACS, and the CFU-GM clonogenic progenitors were enumerated in an in vitro colony assay. *n* = 6 mice in each group, the data are presented as means ± SD; *p ≤ 0.05, **p ≤ 0.01; ***p ≤ 0.001. **B** Lethally irradiated WT mice were transplanted with BMMNCs from WT animals, Nox2-KO mice, and Nox2-KO mice with additional cell treatment with nigericin. 12 days after transplantation, femoral BMMNCs were harvested and plated to count the number of CFU-GM colonies. The spleens were removed and fixed in Fekete’s solution to count the CFU-S colonies. *n* = 6 mice in each group, the data are presented as means ± SD; *p ≤ 0.05; **p ≤ 0.01; ***p ≤ 0.001

To confirm the role of the Nlrp3 inflammasome involvement in homing/engraftment of Nox2-KO HSPCs into WT mice, we exposed mice before transplantation to the Nlrp3 activator nigericin (Fig. 7). As expected, direct stimulation of the Nlrp3 inflammasome by nigericin ameliorated the homing and engraftment defect seen in WT mice transplanted with Nox2-KO BMMNCs.

## Discussion

The seminal observation of this work is that Nox2, by increasing the intracellular level of ROS, activates the Nlrp3 inflammasome, which results in enhanced trafficking of HSPCs. Our study performed using Nox2-KO mice and ROS inhibitor NAC revealed that this occurs by promoting the formation of membrane lipid rafts (MLRs). To support this notion, we demonstrated in the past, for example, SDF-1-receptor CXCR4 [20], an essential role of MLRs in HSPC trafficking. CXCR4 being incorporated on HSPCs into MLRs activated much better downstream signaling in response to the significant BM chemoattractant SDF-1 [20]. As current studies indicate, this observation applies to other “raftophilic receptors.” Overall, we confirm the critical role of Nlrp3 inflammasome in the trafficking of HSPCs and explain this by the involvement of the Nox2-ROS-Nlrp3 inflammasome axis in this process.

Expression of the Nlrp3 inflammasome has been primarily described in monocytes, macrophages, granulocytes, dendritic cells, and lymphocytes [21, 22]. Nlrp3 inflammasome, as a multiprotein intracellular pattern recognition receptor (PRR), consists of Nlrp3 protein, the apoptosis-associated speck-like protein containing a CARD (ASC), and pro-caspase 1 [21]. It is located in the cytoplasm in an inactive form and upon activation, as seen, for example, after administration of pro-mobilizing drugs such as G-CSF or AMD3100, becomes an aggregate composed of several Nlrp3 molecules (speck complexes), each containing Nlrp3 protein, ASC, and pro-caspase 1 [21]. The functional Nlrp3 inflammasome has been recently demonstrated by others [22] and our group [23] in murine and human HSPCs. Moreover, Nlrp3 inflammasome becomes activated not only in HSPCs but also in the BM microenvironment after conditioning for transplantation by lethal irradiation [6]. Important triggers of activation of this PRR are Nox2-derived ROS [24].

Nox2, as a source of ROS, has been implicated in promoting the migration of HSPCs by involving different mechanisms. For example, it has been demonstrated in one of the elegant papers that SDF-1, in response to G-CSF administration, may upregulate hepatocyte growth factor (HGF) that, by interacting with c-Met receptor induces mTOR signaling, leading to FOXO3a inhibition and increased ROS production to promote egress of HSPCs out of the BM into PB [7]. Another proposed mechanism directing the egress of HSPCs from BM involves ROS in promoting sphingosine-1 phosphate (S1P) release that, via its specific receptor S1P_1_R, leads to SDF-1 release from BM stroma cells [8]. Another evidence for the role of Nox2-ROS signaling in the mobilization of HSPCs is data from patients with chronic granulomatous disease (CGD) in which either Nox2 deficiency or mutations in the NADPH oxidase complex subunits render them poor mobilizers [20]. Finally, it has been shown in a model of hind limb ischemia tissue damage that the Nox2-ROS axis plays a critical role in regulating hypoxia and proteolytic activities in BM microenvironment that promote progenitor cell expansion and reparative mobilization of these cells from BM for postischemic tissue repair [25].

Our current work focuses on the molecular explanation of these phenomena. Firstly, we noticed that Nox2-KO mice have lower expression of genes encoding the Nlrp3 inflammasome complex that orchestrates the trafficking of HSPCs. Activation of the inflammasome results in activation of caspase-1, which cleaves pro-interleukin-1β into functional cytokine secreted from the cells. This is a marker of Nlrp3 inflammasome activation. Herein, we demonstrated at the protein level that the Nlrp3 inflammasome is activated at a lower level in Nox2-KO HSPCs.

Our previous work demonstrated that the Nlrp3 inflammasome plays a vital role in regulating the trafficking of HSPCs i) after their pharmacological mobilization into PB [26] as well as ii) in homing and engraftment into BM after hematopoietic transplantation [26]. Accordingly, HSPCs isolated from Nlrp3 inflammasome KO mice perform poorly in Transwell migration assays. Moreover, Nlrp3 inflammasome deficient mice were poor mobilizers, and HSPCs from Nlrp3-KO animals showed defective hematopoietic reconstitution after transplantation into normal controls. To support this data, Nlrp3-KO mice have significantly reduced basic levels of mRNA for SDF-1 in BM stroma cells [26], and we observed the same in a current study in the case of Nox2-KO animals.

Moreover, we report that defective migration of Nox2-KO HSPCs, similar to that for Nlrp3 inflammasome mutant mice, depends on the impaired formation of MLRs on outer cell membranes [6]. This explains the poor responsiveness of these cells to major HSPCs chemoattractants, including SDF-1, S1P, and eATP, whose receptors are “raftophilic” and, for optimal signaling, have to be incorporated into MLRs [6, 18]. Furthermore, since S1P is a significant chemoattractant involved in the egress of HSPCs during mobilization from BM into PB, impaired signaling from S1P receptor type 1 (S1P_1_R) due to its decreased localization in MLR may contribute to poor egress of Nox2-KO cells from BM into PB in response to G-CSF or AMD3100 administration [6].

Thus, our data further confirms the pivotal role of Nlrp3 inflammasome in HSPCs trafficking, and we expand our previous observations by showing that Nox-2 in a ROS-dependent manner activates Nlrp3 inflammasome in HSPCs. We also demonstrate for the first time a novel role of PGE2 in observed phenomena. PGE2 is known to upregulate the expression of CXCR4 on HSPCs and facilitate their homing and engraftment after transplantation [16, 17]. These observations were reported in animal models and clinical trials [17, 27–30]. Herein, we provide novel evidence that PGE2 enhances the trafficking of HSPCs in a Nox2-ROS-Nlrp3-eATP-dependent manner by incorporating CXCR4 into MLRs.

In conclusion, we provide an alternative explanation for why Nox2 deficiency leads to decreased motility of HSPCs. More importantly, our data further support the role of Nlrp3 inflammasome in stem cell trafficking and present that this cytosolic pattern recognition receptor is a target for Nox2-ROS promoting MLRs formation required for optimal responsiveness of HSPCs to their major chemoattractants. We also identified a novel role of PGE2 in the homing of HSPCs not only due to its upregulation of CXCR4 on the cell surface [17] but also due to promoting its incorporation into MLRs. Further studies are needed to address the potential role of other isoforms of Nox (Nox1-5 and Duox1-2). As reported, Nox4 is involved in the early stages of hematopoietic differentiation from iPSCs, and its activity can modulate the specification of iPSCs into CD34^+^.

## Supplementary Information

Below is the link to the electronic supplementary material.

Supplementary Table 1 (DOCX 13.5 KB)

Supplementary Fig. 1 SDF-1 and CXCR4 mRNAs were expressed in BMMNCs samples obtained from WT and Nox2-KO mice measured by qRT-PCR (Panel A). Results of qRT-PCR were normalized to the β2 microglobulin (β2m) expression levels, and to evaluate the relative expression, a comparative ΔCT method was employed. The data represent the mean value ± SD for 3 independent experiments. *p ≤ 0.05 and #*p* ≤ 0.005. Nox2-KO-derived BM cells show lower reactive oxygen species (ROS) activity than WT BM-derived cells (Panel B). BMMNCs were isolated from femurs and tibias of WT and Nox2-KO mice, stained with the DCFDA dye, and incubated with 1 µM PGE2 for 4 h. The activity of reactive oxygen species (ROS) in unstimulated and PGE2-stimulated cells was measured by FACS. (PPTX 239 KB)

Supplementary Fig. 2 Oxygen consumption rate (OCR) measurements. Mitochondrial activity of Lin− cells from WT and Nox-2-KO mouse bone marrow was measured using a Seahorse XF HS MINI instrument. Panel B compares OCR in cells isolated from WT and Nox-2-KO mice. Panel A compares basal and maximum respiration between cells isolated from WT and Nox-2-KO mice. The data represent the mean value ± SD for 3 independent experiments. Statistical significance is indicated by *p ≤ 0.05 and ***p ≤ 0.001. (PPTX 171 KB)

Supplementary Fig. 3 LL-37 does not increase the chemotactic responsiveness of Nox2-KO BMMNCs to low doses of SDF-1. Chemotactic responsiveness was determined by counting the number of CFU-GM colonies formed from the WT and Nox2-KO BMMNCs migrated to the medium supplemented with SDF-1 (5 ng/ml), LL-37 (2.5 μg/ml) in Transwell migration assay. Results are combined from two independent experiments and showed as mean ± SD; *p ≤ 0.05. (PPTX 50.8 KB)

Supplementary Fig. 4 PGE2 upregulates the expression of mRNA for CXCR4, SDF-1, and Nlrp3 inflammasome components in a ROS-dependent manner. Panel A. Expression of SDF-1, CXCR4, Nlrp3, IL-1β, and IL-18 mRNAs in BMMNCs samples stimulated with PGE2 (1 µM) in the absence or presence of NAC (0.5 µM). Results of qRT-PCR were normalized to the β2 microglobulin (β2m) expression levels, and to evaluate the relative expression, a comparative ΔCT method was employed. The data represent the mean value ± SD for three independent experiments: *p ≤ 0.05 and #*p* ≤ 0.005. Panel B. The negative effect of NAC on PGE2-induced MLRs formation on murine SKL cells. BM-purified SKL cells were exposed to PGE2 (1 µM) or PGE2 (1 µM) + NAC (0.5 µM) and stained with lipid raft marker—cholera toxin subunit B (GM1, FITC), rat anti-mouse CXCR4, and secondary anti-rat antibody (Alexa Fluor 594). Confocal analysis showed lipid raft formation in SKL cells stimulated with PEG2 (left panel) but not in SKL cells stimulated with PEG2 in the presence of NAC (right panel). Representative images are shown. (PPTX 1.30 MB)

Supplementary Fig. 5 Inhibition of ROS by NAC results in defective homing and engraftment of transplanted BMMNC and defect in homing and engraftment of NAC-exposed recipient mice. Lethally irradiated experimental animals were transplanted with BMMNCs alone or treated with NAC (Panel A). Lethally irradiated WT mice and mice treated with NAC were transplanted with BMMNCs from WT mice (Panel B). Homing section—24 h after transplantation, the femoral BMMNCs were harvested, FACS evaluated the number of PKH67 + cells, and the CFU-GM clonogenic progenitors were enumerated in an in vitro colony assay. No colonies were formed in lethally irradiated, untransplanted mice (irradiation control). *p ≤ 0.05. Early engraftment section—12 days after transplantation, femoral BMMNCs were harvested and plated to count the number of CFU-GM colonies, and the spleens were removed to count the number of CFU-S colonies. No colonies were formed in lethally irradiated, untransplanted mice (irradiation control). *p ≤ 0.05. Recovery section—White blood cells (WBC) and platelets (PLT) were counted at intervals (at 0, 3, 7, 14, 21, and 28 days after transplantation) in transplanted animals. *p ≤ 0.05; *n* = 9 animals in each group and experiment; results are shown as mean ± SD. (PPTX 142 KB)

## Data Availability

Detailed data is available upon request.
