# Supplementary material for: Defect in Migration of HSPCs in Nox-2 Deficient Mice Explained by Impaired Activation of Nlrp3 Inflammasome and Impaired Formation of Membrane Lipid Rafts
Source: Stem Cell Rev Rep. 2024 Aug 13;21(1):45–58. doi: 10.1007/s12015-024-10775-7 (PMC11762604; doi:10.1007/s12015-024-10775-7)
Supplement: Supplementary file 1 — (DOCX 13.5 KB) [file 12015_2024_10775_MOESM1_ESM.docx]

Table I. **Sequence of murine inflammasome genes primers employed in RT-qPCR.**

| **Gene name** | **Murine** | |
| --- | --- | --- |
|  | **Forward primer sequence** | **Reverse primer sequence** |
| **SDF-1** | F: CGTGAGGCCAGGGAAGAGT | R: TGATGAGCATGGTGGGTTGA |
| **CXCR4** | F: GACCGCCTTTACCCCGATAG | R: GCAGGACGAGACCCACCAT |
| **Nlrp3** | F: GCTGCTGAAGATGACGAGTG | R: TTTCTCGGGCGGGTAATCTT |
| **AIM2** | F: AAAACTGCTCTGCTGCCTCT | R: GATGGCTTCCTGTTCTGCCA |
| **Caspase 1** | F: CACAGCTCTGGAGATGGTGA | R: GGTCCCACATATTCCCTCCT |
| **IL-1β** | F: TCACAGCAGCACATCAACAA | R: TGTCCTCATCCTGGAAGGTC |
| **IL-18** | F: ACAACTTTGGCCGACTTCAC | R: GGGTTCACTGGCACTTTGAT |
| **β-2micro-globulin** | F: CTGACCGGCCTGTATGCTAT | R: CCGTTCTTCAGCATTTGGAT |
