# Supplementary figures and images for: Defect in Migration of HSPCs in Nox-2 Deficient Mice Explained by Impaired Activation of Nlrp3 Inflammasome and Impaired Formation of Membrane Lipid Rafts
Source: Stem Cell Rev Rep. 2024 Aug 13;21(1):45–58. doi: 10.1007/s12015-024-10775-7 (PMC11762604; doi:10.1007/s12015-024-10775-7)

## Slide 1
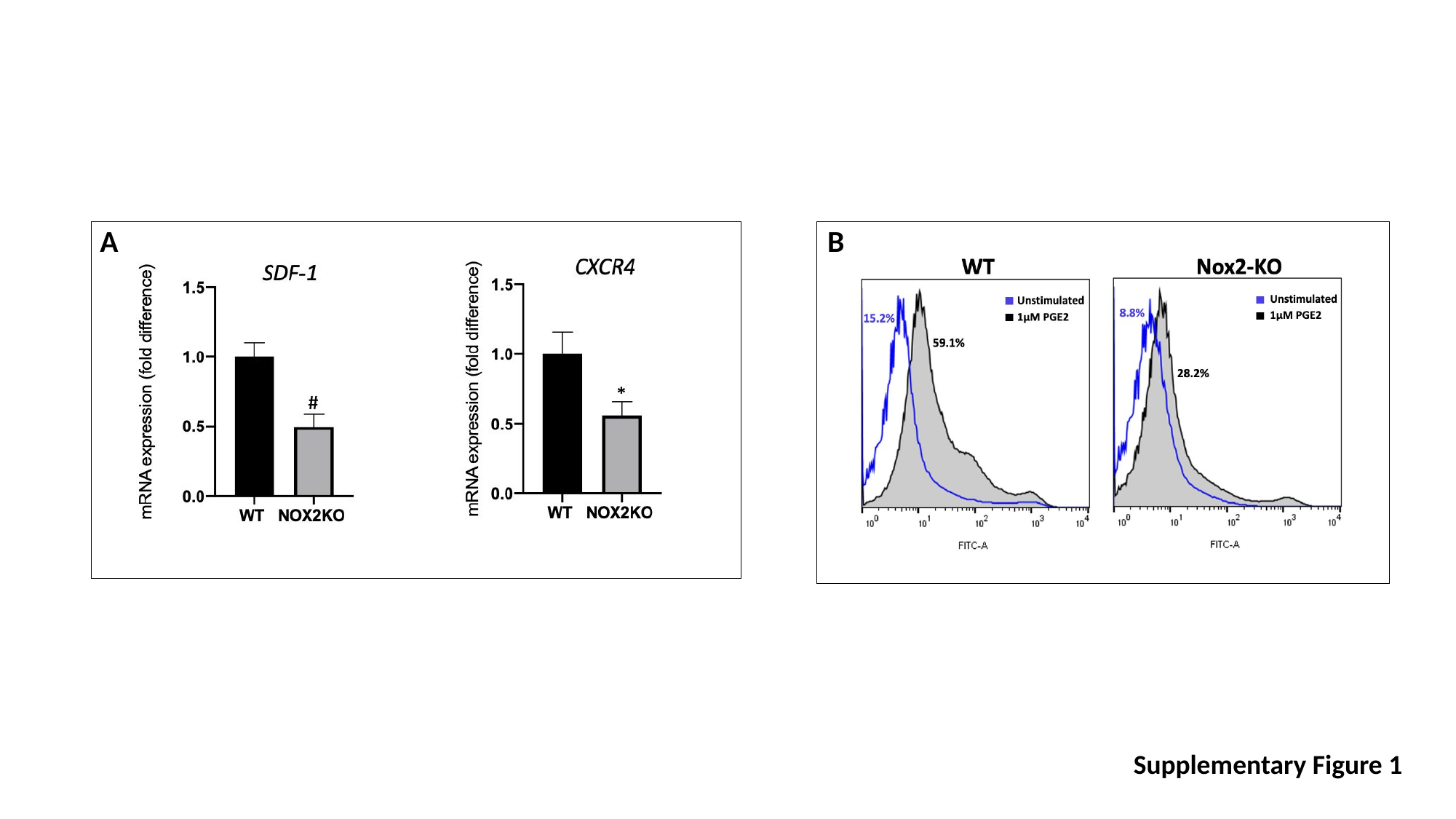

A
B
Supplementary Figure 1

Supplement: Supplementary file 2 — SDF-1 and CXCR4 mRNAs were expressed in BMMNCs samples obtained from WT and Nox2-KO mice measured by qRT-PCR (Panel A). Results of qRT-PCR were normalized to the β2 microglobulin (β2m) expression levels, and to evaluate the relative expression, a comparative ΔCT method was employed. The data represent the mean value ± SD for 3 independent experiments. *p ≤ 0.05 and #p ≤ 0.005. Nox2-KO-derived BM cells show lower reactive oxygen species (ROS) activity than WT BM-derived cells (Panel B). BMMNCs were isolated from femurs and tibias of WT and Nox2-KO mice, stained with the DCFDA dye, and incubated with 1 µM PGE2 for 4 h. The activity of reactive oxygen species (ROS) in unstimulated and PGE2-stimulated cells was measured by FACS. (PPTX 239 KB) [file 12015_2024_10775_MOESM2_ESM.pptx]

## Slide 1
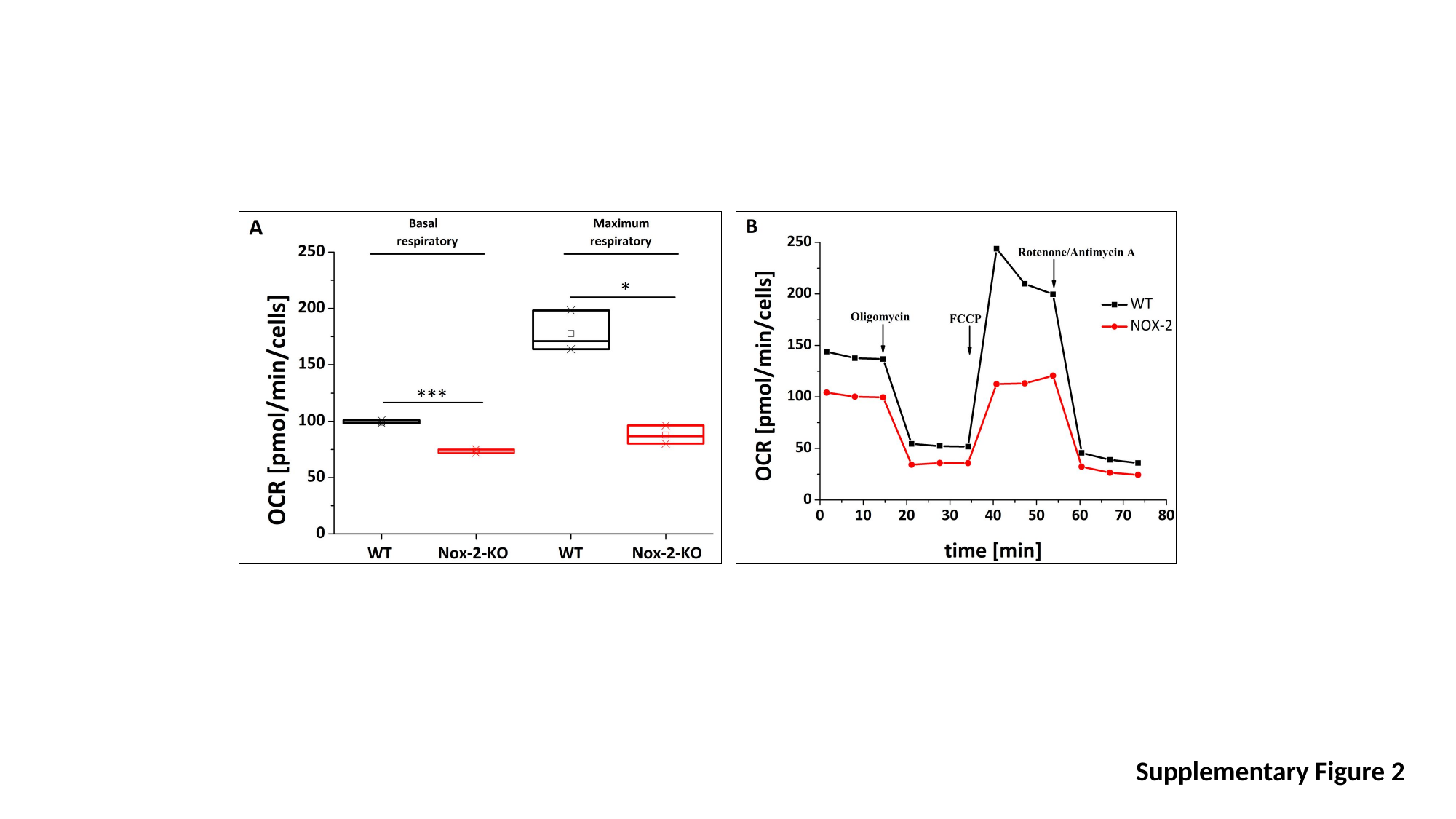

Supplementary Figure 2

Supplement: Supplementary file 3 — Oxygen consumption rate (OCR) measurements. Mitochondrial activity of Lin− cells from WT and Nox-2-KO mouse bone marrow was measured using a Seahorse XF HS MINI instrument. Panel B compares OCR in cells isolated from WT and Nox-2-KO mice. Panel A compares basal and maximum respiration between cells isolated from WT and Nox-2-KO mice. The data represent the mean value ± SD for 3 independent experiments. Statistical significance is indicated by *p ≤ 0.05 and ***p ≤ 0.001. (PPTX 171 KB) [file 12015_2024_10775_MOESM3_ESM.pptx]

## Slide 1
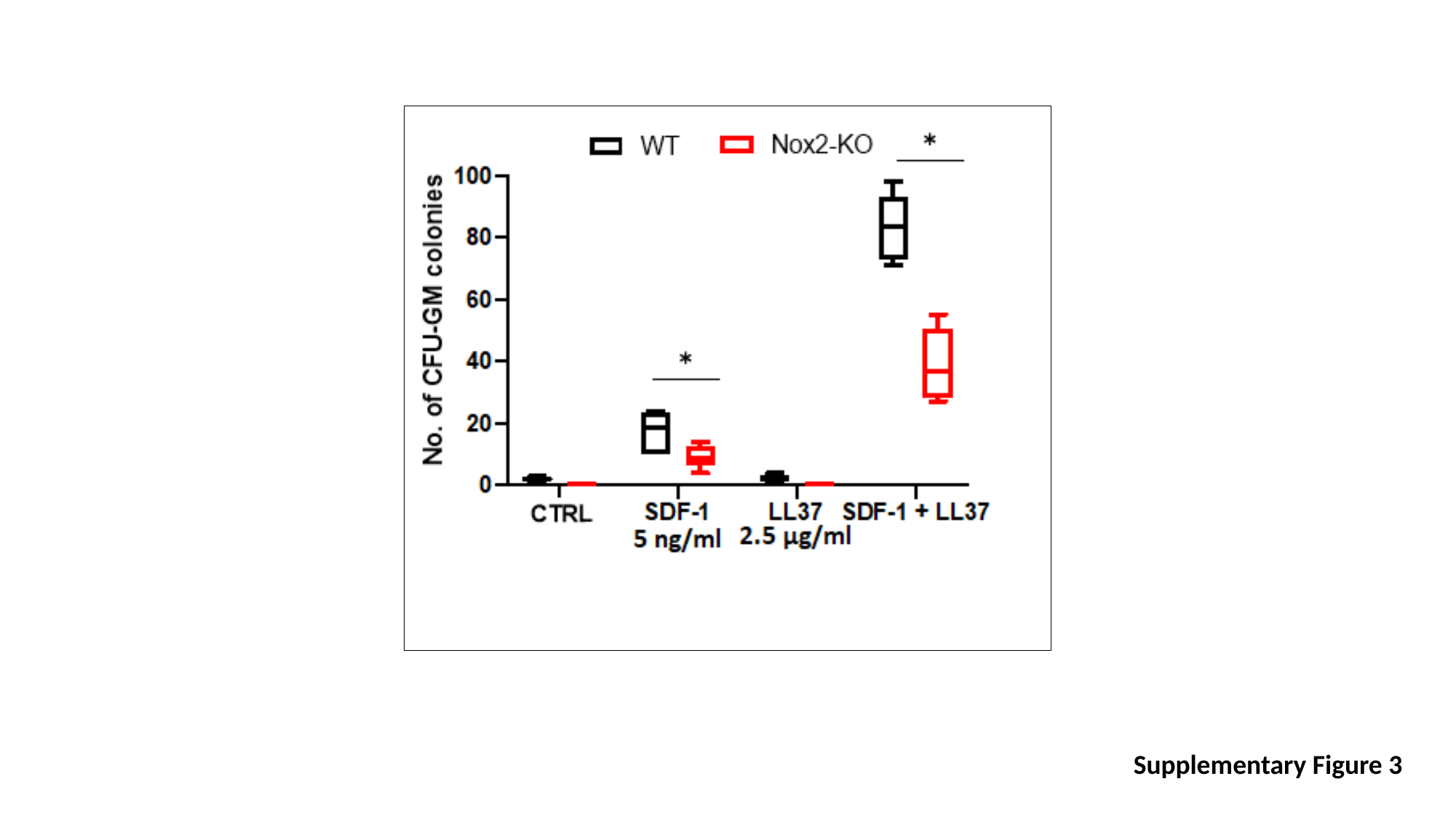

Supplementary Figure 3

Supplement: Supplementary file 4 — LL-37 does not increase the chemotactic responsiveness of Nox2-KO BMMNCs to low doses of SDF-1. Chemotactic responsiveness was determined by counting the number of CFU-GM colonies formed from the WT and Nox2-KO BMMNCs migrated to the medium supplemented with SDF-1 (5 ng/ml), LL-37 (2.5 μg/ml) in Transwell migration assay. Results are combined from two independent experiments and showed as mean ± SD; *p ≤ 0.05. (PPTX 50.8 KB) [file 12015_2024_10775_MOESM4_ESM.pptx]
