# Supplementary material for: Defect in Migration of HSPCs in Nox-2 Deficient Mice Explained by Impaired Activation of Nlrp3 Inflammasome and Impaired Formation of Membrane Lipid Rafts
Source: Stem Cell Rev Rep. 2024 Aug 13;21(1):45–58. doi: 10.1007/s12015-024-10775-7 (PMC11762604; doi:10.1007/s12015-024-10775-7)
Supplement: Supplementary file 5 — PGE2 upregulates the expression of mRNA for CXCR4, SDF-1, and Nlrp3 inflammasome components in a ROS-dependent manner. Panel A. Expression of SDF-1, CXCR4, Nlrp3, IL-1β, and IL-18 mRNAs in BMMNCs samples stimulated with PGE2 (1 µM) in the absence or presence of NAC (0.5 µM). Results of qRT-PCR were normalized to the β2 microglobulin (β2m) expression levels, and to evaluate the relative expression, a comparative ΔCT method was employed. The data represent the mean value ± SD for three independent experiments: *p ≤ 0.05 and #p ≤ 0.005. Panel B. The negative effect of NAC on PGE2-induced MLRs formation on murine SKL cells. BM-purified SKL cells were exposed to PGE2 (1 µM) or PGE2 (1 µM) + NAC (0.5 µM) and stained with lipid raft marker—cholera toxin subunit B (GM1, FITC), rat anti-mouse CXCR4, and secondary anti-rat antibody (Alexa Fluor 594). Confocal analysis showed lipid raft formation in SKL cells stimulated with PEG2 (left panel) but not in SKL cells stimulated with PEG2 in the presence of NAC (right panel). Representative images are shown. (PPTX 1.30 MB) [file 12015_2024_10775_MOESM5_ESM.pptx]

## Slide 1
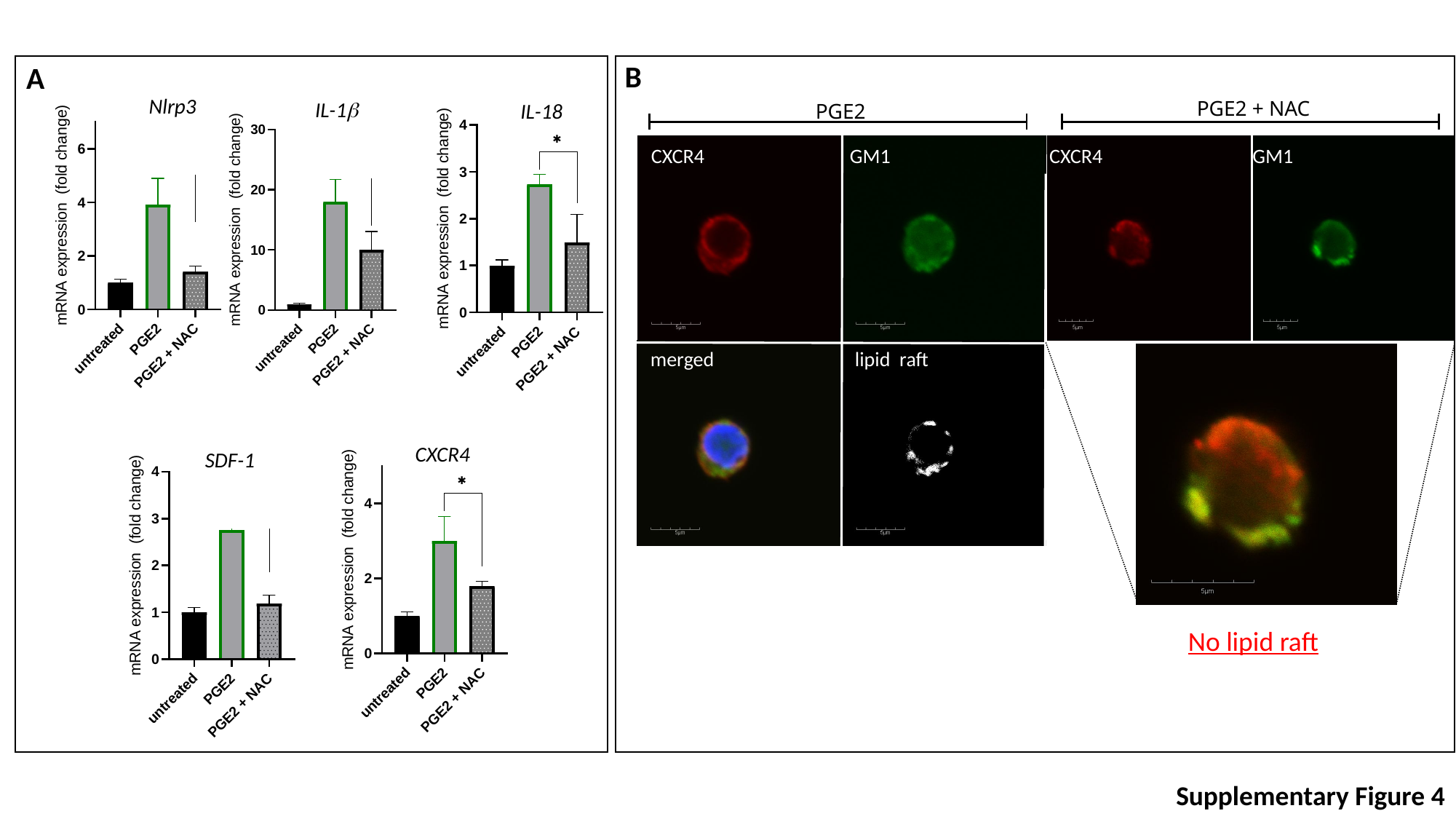

B
PGE2 + NAC
PGE2
merged lipid raft
CXCR4 GM1 CXCR4 GM1
No lipid raft
A
Nlrp3
IL-1b
 IL-18
CXCR4
SDF-1
Supplementary Figure 4
