# Supplementary material for: Defect in Migration of HSPCs in Nox-2 Deficient Mice Explained by Impaired Activation of Nlrp3 Inflammasome and Impaired Formation of Membrane Lipid Rafts
Source: Stem Cell Rev Rep. 2024 Aug 13;21(1):45–58. doi: 10.1007/s12015-024-10775-7 (PMC11762604; doi:10.1007/s12015-024-10775-7)
Supplement: Supplementary file 6 — Inhibition of ROS by NAC results in defective homing and engraftment of transplanted BMMNC and defect in homing and engraftment of NAC-exposed recipient mice. Lethally irradiated experimental animals were transplanted with BMMNCs alone or treated with NAC (Panel A). Lethally irradiated WT mice and mice treated with NAC were transplanted with BMMNCs from WT mice (Panel B). Homing section—24 h after transplantation, the femoral BMMNCs were harvested, FACS evaluated the number of PKH67 + cells, and the CFU-GM clonogenic progenitors were enumerated in an in vitro colony assay. No colonies were formed in lethally irradiated, untransplanted mice (irradiation control). *p ≤ 0.05. Early engraftment section—12 days after transplantation, femoral BMMNCs were harvested and plated to count the number of CFU-GM colonies, and the spleens were removed to count the number of CFU-S colonies. No colonies were formed in lethally irradiated, untransplanted mice (irradiation control). *p ≤ 0.05. Recovery section—White blood cells (WBC) and platelets (PLT) were counted at intervals (at 0, 3, 7, 14, 21, and 28 days after transplantation) in transplanted animals. *p ≤ 0.05; n = 9 animals in each group and experiment; results are shown as mean ± SD. (PPTX 142 KB) [file 12015_2024_10775_MOESM6_ESM.pptx]

## Slide 1
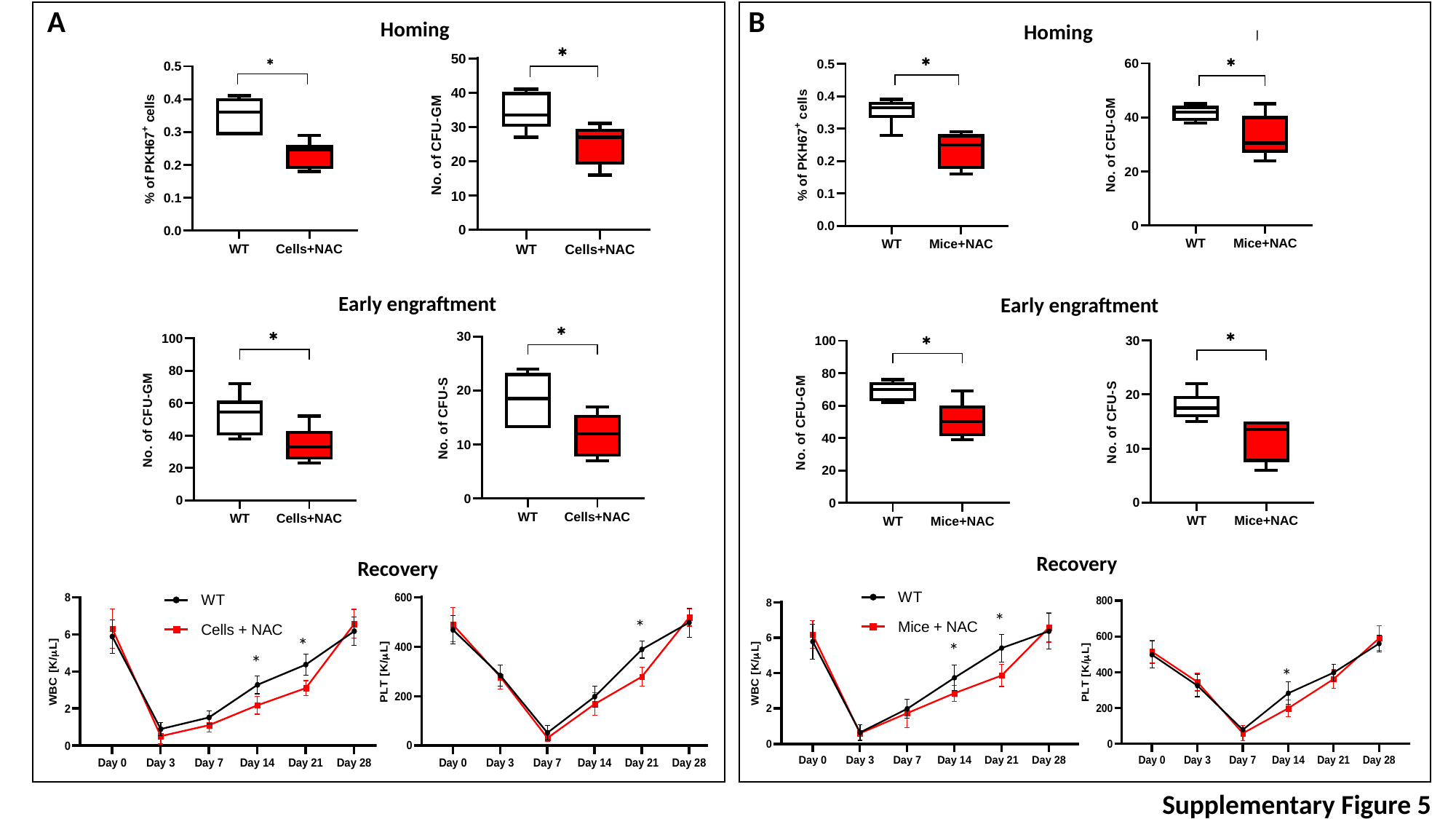

A
Homing
Early engraftment
Recovery
*
*
*
B
Homing
Early engraftment
Recovery
*
*
*
Supplementary Figure 5
